# Epigenetic regulation of diverse cell death modalities in cancer: a focus on pyroptosis, ferroptosis, cuproptosis, and disulfidptosis

**DOI:** 10.1186/s13045-024-01545-6

**Published:** 2024-04-23

**Authors:** Shimeng Zhou, Junlan Liu, Andi Wan, Yi Zhang, Xiaowei Qi

**Affiliations:** 1 Department of Breast and Thyroid Surgery, Southwest Hospital, Army Medical University, Chongqing, China; 2 Key Laboratory of Chongqing Health Commission for Minimally Invasive and Precise Diagnosis, Chongqing, China

**Keywords:** Epigenetic modification, Pyroptosis, Ferroptosis, Cuproptosis, Disulfidptosis

## Abstract

Tumor is a local tissue hyperplasia resulted from cancerous transformation of normal cells under the action of various physical, chemical and biological factors. The exploration of tumorigenesis mechanism is crucial for early prevention and treatment of tumors. Epigenetic modification is a common and important modification in cells, including DNA methylation, histone modification, non-coding RNA modification and m6A modification. The normal mode of cell death is programmed by cell death-related genes; however, recent researches have revealed some new modes of cell death, including pyroptosis, ferroptosis, cuproptosis and disulfidptosis. Epigenetic regulation of various cell deaths is mainly involved in the regulation of key cell death proteins and affects cell death by up-regulating or down-regulating the expression levels of key proteins. This study aims to investigate the mechanism of epigenetic modifications regulating pyroptosis, ferroptosis, cuproptosis and disulfidptosis of tumor cells, explore possible triggering factors in tumor development from a microscopic point of view, and provide potential targets for tumor therapy and new perspective for the development of antitumor drugs or combination therapies.

**Supplementary Information:**

The online version contains supplementary material available at 10.1186/s13045-024-01545-6.

## Introduction

Cancer, the leading cause of global human death, refers to the proliferation of local histiocytes caused by some tumorigenic factors. The whole process is generally divided into four stages: precancerous lesion, carcinoma in situ, invasive carcinoma and metastasis [1, 2]. Cancer represents a complex ecosystem, including cancer cells and non-cancer cells. Various cell-cell interactions form the tumor microenvironment (TME), which includes immune cells, cancer-associated fibroblasts, and endothelial cells [2]. TME is involved in biological functions of cancer, such as maintaining proliferation, inducing angiogenesis, activating invasion and metastasis, and promoting inflammation [3]. Non-physiological death of cancer cells affects tumor progression.

Cell death has physiological and pathological functions. However, infection, chronic inflammation and tissue damage lead to sudden death of a large number of cells and then the release of large amount of cellular content into the extracellular space causes damage to the organ [4]. Cell death can be induced by genetically programmed suicide mechanisms such as apoptosis, necrotic apoptosis and pyroptosis, or it can be attributed to metabolic dysregulation, such as ferroptosis, cuproptosis, disulfidptosis [5]. In recent years, cellular pyroptosis, ferroptosis, cuproptosis, and disulfidptosis have received increasing attention, especially for their effects on tumor progression. Pyroptosis is a mode of programmed cell death in which inflammatory vesicles activated by intracellular and extracellular stimuli lead to the rupture of cell membrane, followed by the release of cellular content. Ferroptosis and cuproptosis are mainly due to an imbalance in Fe^2+^ and Cu^+^ homeostasis in the body, thereby altering a series of cell signaling pathways that play a direct or indirect role in cancer. Disulfidptosis is a new form of cell death caused by massive accumulation of intracellular disulfide molecules triggered by increased cystine level and the following NADPH depletion [6].

The imbalance between substantial cell death and poor cell proliferation or recruitment leads to the change of cell number, which is helpful to study the pathogenesis of cancer. The cell death patterns are the key to explore the pathogenesis of cancer. Under normal circumstances, the cells are in homeostasis; the aging or damaged cells are cleared by programmed cell death, which is usually achieved through cell apoptosis. However, in the early stage of cell death, cells are stimulated by internal and external factors, inducing regulatory necrosis of cells such as pyroptosis, ferroptosis, cuproptosis, and disulfidptosis [7].The patients receiving treatment in early stage of cancer show a better prognosis compared with those treated at advanced stage; therefore, it is essential to clarify the mechanism of cancer onset. This paper focuses on the mechanism of early cell death, such as pyroptosis, ferroptosis, cuproptosis, and disulfidptosis, providing new perspectives for the development of antitumor drugs or combination therapies.

Various modes of cell death can play important roles in regulating tumor development, progression and drug resistance through different signaling pathways. Epigenetic modifications is involved in the regulation of multiple biological processes and signaling pathways. Therefore, we focus on epigenetic regulation to investigate the effects of different cell death modes on tumor progression and treatment, which can provide a theoretical basis for antitumor strategies and give a clue to new therapeutic markers.

## Epigenetic modifications

**Fig. 1:**
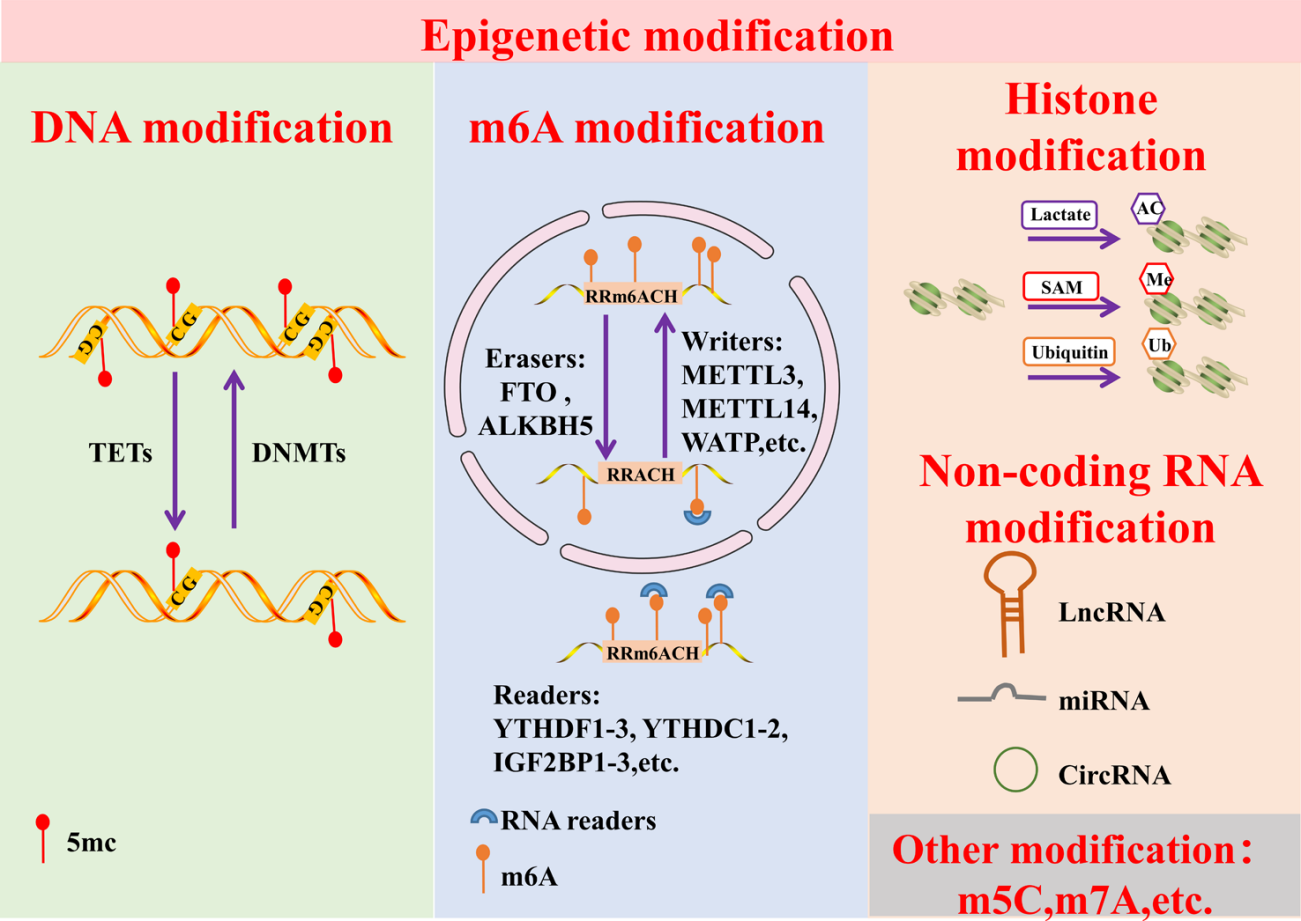
Classification of epigenetic modifications. Epigenetic modifications are divided into the following main types. First, DNA methylation modification. DNA methylation modification is mainly regulated by two types enzymes involved, tet methylcytosine dioxygenases (TETs), which is responsible for DNA demethylation, and DNA methyltransferases (DNMTs), which is responsible for DNA methylation. Second, m6A modification. m6A modification consists of three enzymes acting in combination with each other. Respectively, eraser: Fat mass and obesity associated (FTO) and AlkB homolog 5, RNA demethylase (ALKBH5). Writer: methyltransferase 3 (METTL3), methyltransferase 14 (METTL14) and so on. Reader: YTH N6-methyladenosine RNA binding protein F1/2/3 (YTHDF1/2/3), YTH N6-methyladenosine RNA binding protein C1/2/3 (YTHDC1/2/3) and insulin like growth factor 2 mRNA binding protein 1/2/3 (IGF2BP1/2/3). Third, histone modification, including histone acetylation, histone methylation, and histone ubiquitination. In addition, there are non-coding RNA modifications, including miRNAs, lncRNAs, and circRNA

Epigenetics is defined as the study of heritable changes that cannot be attributed to DNA sequence variation [8]. Epigenetic modifications includes DNA methylation, histone modification, chromatin remodeling, non-coding RNA (ncRNA), and RNA methylation [9](Fig. 1). DNA methylation usually occurs at cytosines that precede a guanine nucleotide or CpG sites, and abnormal DNA methylation can serve as a diagnostic and prognostic marker [10, 11]. The basic unit of chromatin is the nucleosome consisting histones H2A, H2B, H3 and H4, which are heavily post-translationally modified in several ways, including phosphorylation, ubiquitination, acetylation and methylation [12]. Histone modification refers to the post-translational modification (PTM) of amino acids in the N-terminus of histones, including histone acetylation, histone methylation, and histone ubiquitination [13]. Histone modification affects the development and prognosis of lung cancer [14], glioma [15], breast cancer [16], peripheral T-cell lymphoma [17], and rectal cancer [18]. In addition, the remodeling of damaged chromatin predicts a higher survival rate of advanced biliary tract cancer [19]. JAK-STAT signaling pathway-mediated chromatin remodeling affects drug resistance in lymphoma [20]. Non-coding RNA, including miRNA, lncRNA and circRNA, participates in the regulation of TME [21].

RNA modifications promote or inhibit tumorigenesis by regulating cell proliferation, differentiation, invasion, migration, stemness, metabolism and drug resistance. Among numerous RNA modifications, N6-methyladenosine (m6A), 5-methylcytidine (m5C), and N7-methylguanosine (m7G) have been associated with tumorigenesis [22]. Defined as the most common internal modification in mRNA and long non-coding RNA (lncRNA), m6A methylation often occurs in 3′untranslated regions and affects relevant physiological functions, pathological processes, and tumorigenesis [23]. It is regulated by three elements: methyltransferases (“writers”), such as methyltransferase-like protein 3 (METTL3), METTL14, WATP and RBM15; RNA-binding proteins (“readers”), such as the YTH domain family protein (YTHDF) 1/2/3, YTH domain-containing protein (YTHDC) 1/2/3, and IGF2BP1/2/3; and demethylases (“erasers”), such as fat mass and obesity-associated protein (FTO) and AlkB homolog 5 (ALKBH5) [24]. m6A methylation is involved in the tumorigenesis and progression of gastric cancer (GC) [25], bladder cancer (BC) [26], ovarian cancer [27], and hepatocellular carcinoma (HCC) and so on [28]. However, whether m6A methylation exhibits anti-tumor or tumor-promoting effects is still controversial [29].

m5C modification is the methylation of the fifth C atom of RNA cytosine. It is found in a wide variety of RNA molecules, including tRNA, rRNA, mRNA and ncRNA. m5C RNAs can maintain RNA stability and regulate protein synthesis and translation [30]. m5C is also regulated by “writers”, “erasers”, and “readers”. m5C methyltransferases “writers” include the NSUNs family and DNMTs family. m5C demethyltransferases “erasers” are catalyzed by the TETs family. The “readers” include Aly/REF nuclear export factor (ALYREF) and Y-box binding protein 1 (YBX1) [22]. The level of m5C is closely related to tumorigenesis. NSUN2 promotes the proliferation, migration and invasion of GC cells through up-regulation of m5C [31]. In non-small cell lung cancer (NSCLC), aberrant m5C hypermethylation mediates the resistance to gefitinib via the NSUN2/YBX1/QSOX1 axis [32].

m7G, a methylation of guanine N7, adds a positive charge to the N atom, affecting the structure of RNA through electrostatic and spatial effects. m7G-cap is important for mRNA shearing and processing, nucleoplasmic translocation and protein synthesis [22]. Abnormal m7G levels are closely associated with tumorigenesis and progression by regulating the expression of a variety of oncogenes. m7G “readers” include three protein complexes: RNMT/RAM, METTL1/WDR4 and WBSCR22/TRMT112. m7G “readers” include eukaryotic translation initiation factor 4E (eIF4E) family proteins (eIF4E1/eIF4E, eIF4E2/4EHP, and eIF4E3), and Ago2 [33]. Studies have shown that m7G modification is significantly associated with tumorigenesis and progression of cancers. In a variety of cancers, m7G modification promotes cancer cell growth, proliferation and tumor formation through the METTL1/WDR4/Arg-TCT-4-1 axis [34]. In addition, the METTL1-m7G-EGFR/EFEMP1 axis promotes the development of BC, leading to a poor prognosis of patients [35]. m7G modification promotes the development of oesophageal squamous cell carcinoma via the RPTOR/ULK1/autophagy axis [36]. Since epigenetic modification is significantly associated with tumorigenesis and progression, we want to explore whether epigenetic modifications affect different modes of cell death and thus participate in the regulation of tumor progression and chemoresistance.

## Molecular mechanisms of different cell death pathways

### Pyroptosis

**Fig. 2:**
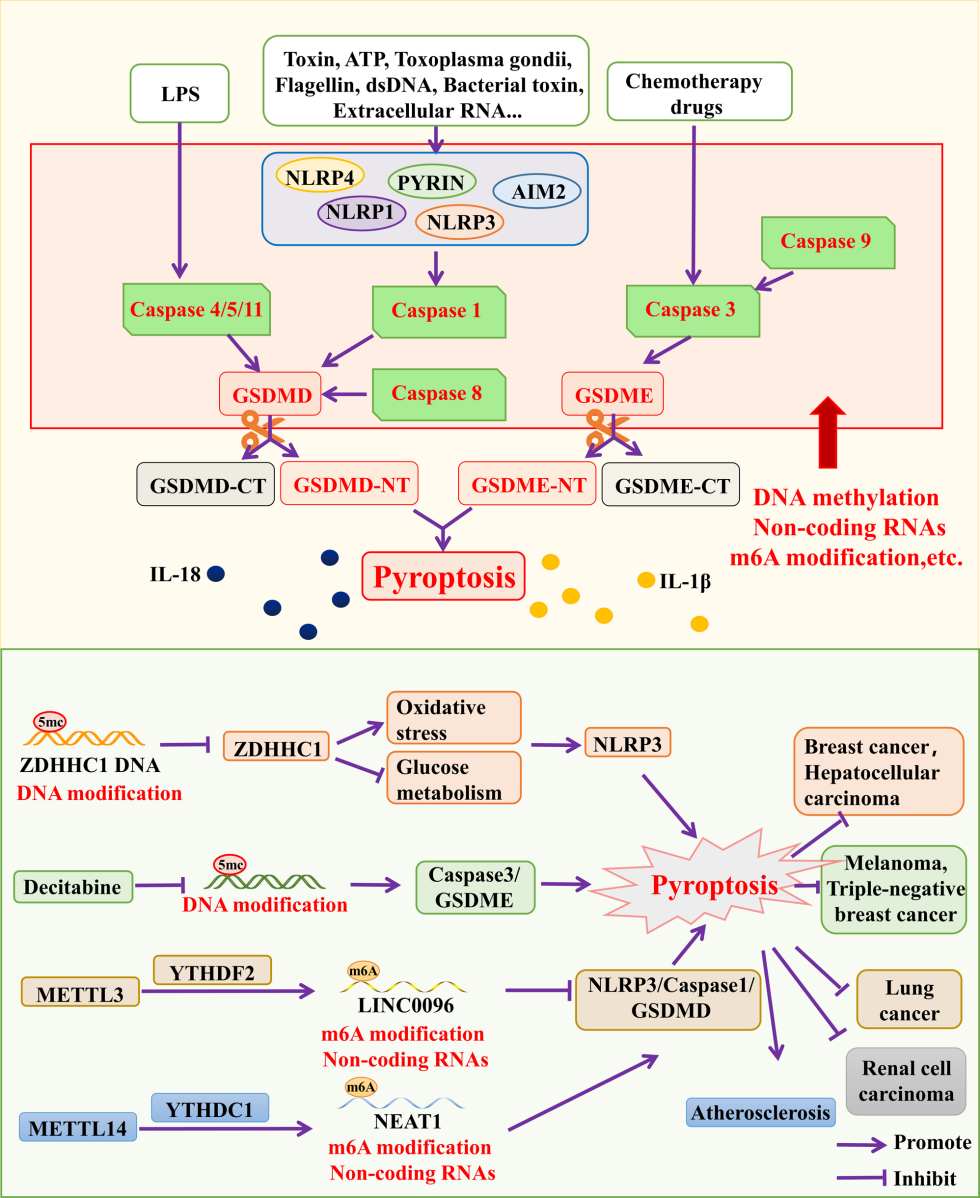
Mechanisms of cellular pyroptosis. Various factors stimulate inflammasomes, which then induce pyroptosis by cleavage of GSDMD and GSDME by the caspase family, releasing IL-18 and IL-1β. *LPS* Lipopolysaccharide, *NLRP1* NLR family pyrin domain containing (1), *NLRP3* NLR family pyrin domain containing 3, *NLRP4* NLR family pyrin domain containing 4, *AIM2* Absent in melanoma (2), *PYRIN* MEFV innate immunity regulator, pyrin, *GSDMD* Gasdermin D, *GSDME* Gasdermin E, *IL-18* Interleukin 18, *IL-1β* Interleukin 1β

With the development of cellular study, recent studies have found more novel modes of cell death, like pyroptosis, ferroptosis, cuproptosis, and disulfidptosis. In order to investigate the mechanism of tumor development and find therapeutic targets, researchers pay more attention to these new death modes of tumor cells. Pyroptosis is a kind of programmed cell death in which continuous expansion of cells results in the membrane​ rupture and the contents release. It is involved in a variety of physiological and pathological processes mainly by activating a strong inflammatory response [37]. There are five major inflammasomes in the pyroptosis pathway: NLRP3, AIM2, NLRP1, PYRIN, and NLRC4 [37]. The inflammasomes activate caspase-1 and cleave GSDMD, usually the N-terminal domain of GSDMD, thus induce pyroptosis [38]. Furthermore, caspase-8 and caspase-3 can directly cleave GSDMD to induce pyroptosis [39] (Fig. 2).

Substantial studies have shown that pyroptosis can promote or inhibit the development and metastasis of many cancers, such as GC, BRCA-associated breast cancer, BC, and lung cancer (Table 1). Cucurbitacin B directly binds to TLR4 to activate NLRP3 inflammasome and pyroptosis, thereby playing an anti-tumor role inNSCLC [40]. Cisplatin activates the MEG3/NLRP3/caspase-1/GSDMD pathway to induce pyroptosis in triple-negative breast cancer (TNBC), thereby inhibiting tumor growth and metastasis [41]. Tumor suppressor DRD2 promotes M1 polarization of macrophages and inhibits NF-κB signaling pathway to trigger breast cancer apoptosis, providing a new predictive and therapeutic target for breast cancer [42]. Under hypoxia, the formation of nPD-L1/p-Stat3 complex increases the expression of GSDMC in different cancer cells, converting apoptosis to pyroptosis so as to promote tumor progression and suppress anti-tumor immune responses [43]. Epigenetic modifications can also mediate tumor growth and metastasis by regulating pyroptosis-related pathways.

**Table 1:** Epigenetic regulation of tumor cell pyroptosis

Gene	Title	Epigenetic modulation	Cancer
ZDHHC1	DNA methylation downregulated ZDHHC1 suppresses tumor growth by altering cellular metabolism and inducing oxidative/ER stress-mediated apoptosis and pyroptosis	DNA methylation	Various cancers
GSDME	Antitumor effect of simvastatin in combination with DNA methyltransferase inhibitor on gastric cancer via GSDME-mMediated pyroptosis	DNA methylation	Gastric cancer
GSDME	Programming cell pyroptosis with biomimetic nanoparticles for solid tumor immunotherapy	DNA methylation	Solid tumor
GSDMD	Elevated methionine flux drives pyroptosis evasion in persister cancer Cells	DNA methylation	Persister Cancer
GSDME	Gasdermin E deficiency attenuates acute kidney injury by inhibiting pyroptosis and inflammation	DNA methylation	Colorectal tumours
GSDME	Large-scale analysis of DFNA5 methylation reveals its potential as biomarker for breast cancer	DNA methylation	Breast cancer
GSDME	Postsurgical wound management and prevention of triple-negative breast cancer recurrence with a pryoptosis-inducing, photopolymerizable hydrogel	DNA methylation	Breast cancer
GSDMA/B/D/E	GSDMs are potential therapeutic targets and prognostic biomarkers in clear cell renal cell carcinoma	DNA methylation	Clear cell renal cell carcinoma
GSDMD	Co-delivery of nigericin and decitabine using hexahistidine-metal nanocarriers for pyroptosis-induced immunotherapeutics	DNA methylation	Bladder cancer
Caspase−1	G9A promotes tumor cell growth and invasion by silencing CASP1 in non-small-cell lung cancer cells	Histone modification	Lung cancer
Caspase−1	PRMT5 regulates cell pyroptosis by silencing CASP1 in multiple myeloma	Histone modification	Multiple myeloma
Caspase−1	Inhibition of BRD4 prevents proliferation and epithelial-mesenchymal transition in renal cell carcinoma via NLRP3 inflammasome-induced pyroptosis	Histone modification	Renal cancer
Caspase−1	Death by histone deacetylase inhibitor quisinostat in tongue squamous cell carcinoma via apoptosis, pyroptosis, and ferroptosis	Histone modification	Tongue cancer
Caspase−1	4,5-Dimethoxycanthin−6-one is a novel LSD1 inhibitor that inhibits proliferation of glioblastoma cells and induces apoptosis and pyroptosis	Histone modification	Glioblastoma
microRNA−125b/Caspase−1	Tanshinone IIA regulates microRNA‑125b/foxp3/caspase‑1 signaling and inhibits cell viability of nasopharyngeal carcinoma	Non-coding RNAs	Nasopharynx cancer
miR−145/GSDMD	Tanshinone II A enhances pyroptosis and represses cell proliferation of HeLa cells by regulating miR−145/GSDMD signaling pathway	Non-coding RNAs	Cervical cancer
miR−182/NLRP3	Stat5 inhibits NLRP3-mediated pyroptosis to enhance chemoresistance of breast cancer cells via promoting miR−182 transcription	Non-coding RNAs	Breast cancer
MiR-495/NLRP3	NLRP3 Inflammasome Activation by MicroRNA−495 Promoter Methylation May Contribute to the Progression of Acute Lung Injury	Non-coding RNAs	Acute lung injury
LncRNA MEG3/NLRP3	Cisplatin Induces Pyroptosis via Activation of MEG3/NLRP3/caspase−1/GSDMD Pathway in Triple-Negative Breast Cancer	Non-coding RNAs	Breast cancer
LncRNA TCONS−14,036/NLRP3	The sodium new houttuyfonate suppresses NSCLC via activating pyroptosis through TCONS−14,036/miR−1228−5p/PRKCDBP pathway	Non-coding RNAs	Lung cancer
METTL3/YTHDF2/NLRP3	LncRNA LINC00969 promotes acquired gefitinib resistance by epigenetically suppressing of NLRP3 at transcriptional and posttranscriptional levels to inhibit pyroptosis in lung cancer	m6A modification	Lung cancer
METTL14/YTHDF2	Methyltransferase-like 14 suppresses growth and metastasis of renal cell carcinoma by decreasing long noncoding RNA NEAT1	m6A modification	Renal cell carcinoma
YTHDF1/GSDMD	YTHDF1 alleviates sepsis by upregulating WWP1 to induce NLRP3 ubiquitination and inhibit caspase−1-dependent pyroptosis	m6A modification	Sepsis
METTL14/TINCR/NLRP3	METTL14 suppresses pyroptosis and diabetic cardiomyopathy by downregulating TINCR lncRNA	m6A modification	Diabetic cardiomyopathy
YTHDC1/ Caspase−1	LncRNA FENDRR with m6A RNA methylation regulates hypoxia-induced pulmonary artery endothelial cell pyroptosis by mediating DRP1 DNA methylation	m6A modification	Hypoxic pulmonary hypertension
METTL3/ Caspase−1	The METTL3/MALAT1/PTBP1/USP8/TAK1 axis promotes pyroptosis and M1 polarization of macrophages and contributes to liver fibrosis	m6A modification	Liver fibrosis
METTL3/ NLRP3	Total Flavones of Abelmoschus manihot Ameliorates Podocyte Pyroptosis and Injury in High Glucose Conditions by Targeting METTL3-Dependent m6A Modification-Mediated NLRP3-Inflammasome Activation and PTEN/PI3K/Akt Signaling	m6A modification	Diabetic nephropathy

### Ferroptosis

**Fig. 3:**
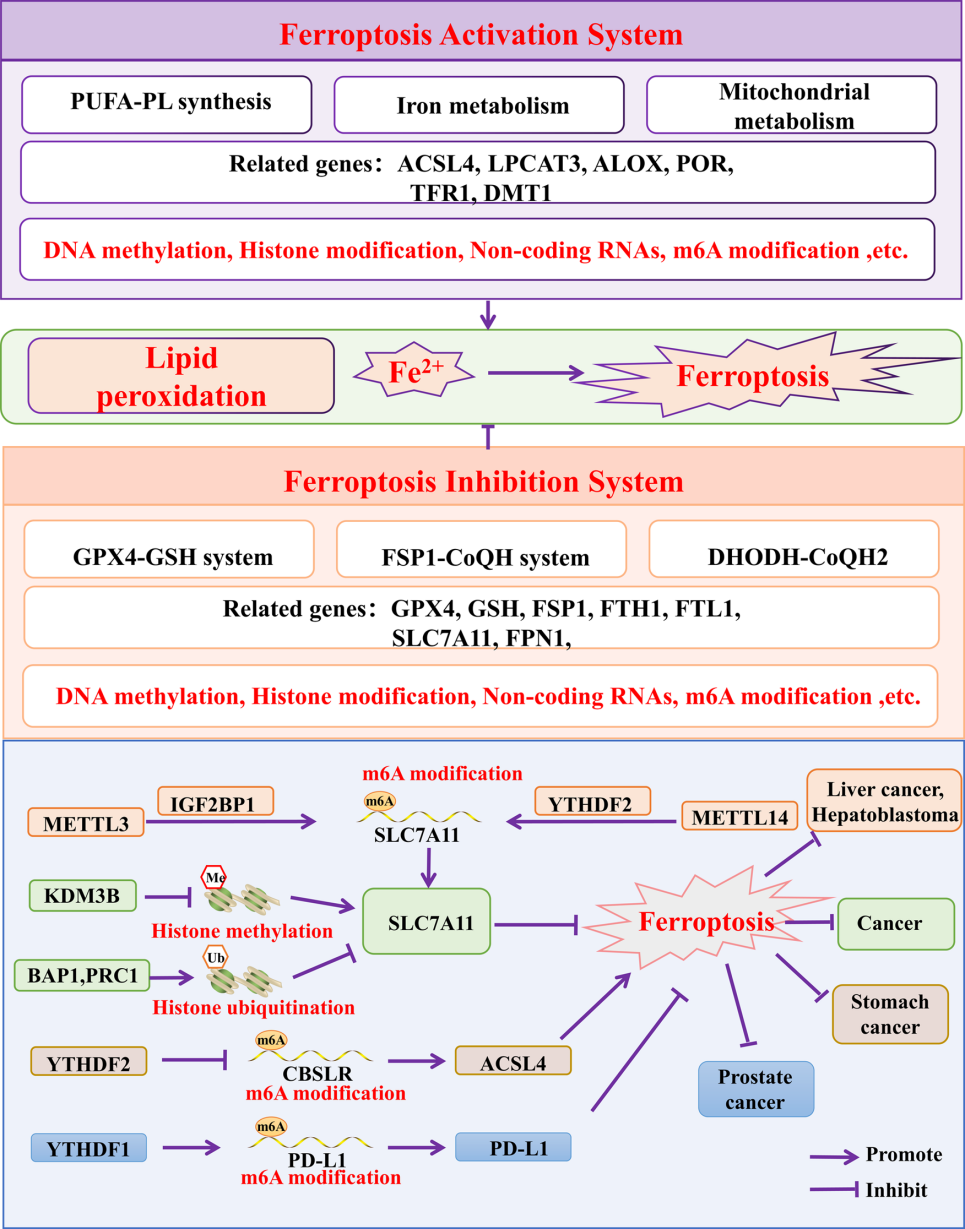
Mechanisms of cellular ferroptosis. Lipid peroxidation is affected when the ferroptosis-activation and ferroptosis-inhibition systems receive a corresponding stimulus. Lipid peroxidation and Fe^2+^ accumulation eventually induce ferroptosis. PUFA-PL: Polyunsaturated fatty acid-containing phospholipid. ACSL4: Acyl-CoA synthetase long chain family member 4. LPCAT3: Lysophosphatidylcholine acyltransferase 3. ALOX: Arachidonate lipoxygenase. POR: Cytochrome p450 oxidoreductase. *TFR1* Transferrin receptor, *DMT1* Ferrous ion membrane transport protein DMT1, *GPX4* Glutathione peroxidase 4, *GSH* Glutathione, *FSP1* Ferroptosis suppressor protein-1, *CoQH2* ubiquinol, *DHODH* Dihydroorotate dehydrogenase, *FTH1* Ferritin heavy chain 1 FTL1: Ferritin light polypeptide 1, *FPN1* Solute carrier family 40 member 1, *SLC7A11* Solute carrier family 7 member 11

Ferroptosis is cell death in which excessive iron ions get into the cell and induce the Fenton reaction, resulting in the modulation of multiple cellular metabolic pathways, such as redox homeostasis disruption, mitochondrial activity, amino acids and lipids metabolism [50, 51]. PUFA-PL synthesis, iron metabolism and mitochondrial metabolism can cause lipid peroxidation to induce ferroptosis. On the contrary, GPX4-GSH system, FSP1-CoQH system and DHODH-CoQH2 system can inhibit lipid formation and thus inhibit ferroptosis [52] (Fig. 3). Evidences in recent years have revealed that ferroptosis is involved in the regulation of cancer, such as GC, glioma, lung squamous cell carcinoma, BRCA-associated breast cancer, BC, and lung cancer (Table 2).

**Table 2:** Epigenetic regulation of tumor cell ferroptosis

Gene	Title	Epigenetic modulation	Cancer
GPX4/ACSL4	Polyunsaturated fatty acid biosynthesis pathway determines ferroptosis sensitivity in gastric cancer	DNA methylation	Gastric cancer
16 CpG sites	Ferroptosis-associated DNA methylation signature predicts overall survival in patients with head and neck squamous cell carcinoma	DNA methylation	Head and neck squamous cell carcinoma
TFRC/ACSL4	ALKBH5 inhibits thyroid cancer progression by promoting ferroptosis through TIAM1-Nrf2/HO−1 axis	DNA methylation	Glioma
ZEB1/DUOX1	Identification and Validation of Ferroptosis-Related DNA Methylation Signature for Predicting the Prognosis and Guiding the Treatment in Cutaneous Melanoma	DNA methylation	Cutaneous Melanoma
SLC7A11/H3K9/H2A	Cystine transporter SLC7A11/xCT in cancer: ferroptosis, nutrient dependency, and cancer therapy	DNA methylation/ Histone modification	Multiple human cancers
NR4A2/GPX4/H2A/ H3/H4 histone	Ferroptosis Induction in Multiple Myeloma Cells Triggers DNA Methylation and Histone Modification Changes Associated with Cellular Senescence	DNA methylation/ Histone modification	Multiple myeloma
OTUB1/SLC7A11	The ubiquitin hydrolase OTUB1 promotes glioma cell stemness via suppressing ferroptosis through stabilizing SLC7A11 protein	Histone modification	Glioma
OTUB1/SLC7A11	The Deubiquitylase OTUB1 Mediates Ferroptosis via Stabilization of SLC7A11	Histone modification	Bladder cancer
H3K9me3/H3K27me3/ SLC7A11	Ferritinophagy is required for the induction of ferroptosis by the bromodomain protein BRD4 inhibitor (+)-JQ1 in cancer cells	Histone modification	Breast cancer
H2B/SLC7A11	Epigenetic regulation of ferroptosis by H2B monoubiquitination and p53	Histone modification	Hepatocellular carcinoma
miR−200 family	Epigenetic reprogramming of epithelial-mesenchymal transition promotes ferroptosis of head and neck cancer	Non-coding RNAs	Head and neck cancer
miR−522/ALOX15	CAF secreted miR−522 suppresses ferroptosis and promotes acquired chemo-resistance in gastric cancer	Non-coding RNAs	Gastric cancer
lncRNA H19/miR−19b−3p/FTH1	Curcumenol triggered ferroptosis in lung cancer cells via lncRNA H19/miR−19b−3p/FTH1 axis	Non-coding RNAs	Lung cancer
miR−545−3p/SLC7A11	Circular RNA Circ_0067934 Attenuates Ferroptosis of Thyroid Cancer Cells by miR−545−3p/SLC7A11 Signaling	Non-coding RNAs	Thyroid Cancer
miR−27a−3p	CircBCAR3 accelerates esophageal cancer tumorigenesis and metastasis via sponging miR−27a−3p	Non-coding RNAs	Esophageal cancer
LINC00336	Long noncoding RNA LINC00336 inhibits ferroptosis in lung cancer by functioning as a competing endogenous RNA	Non-coding RNAs	Lung cancer
ACSL4	Cancer-associated fibroblasts suppress ferroptosis and induce gemcitabine resistance in pancreatic cancer cells by secreting exosome-derived ACSL4-targeting miRNAs	Non-coding RNAs	Pancreatic cancer
lncRNA DACT3-AS1	Loss of cancer-associated fibroblast-derived exosomal DACT3-AS1 promotes malignant transformation and ferroptosis-mediated oxaliplatin resistance in gastric cancer	Non-coding RNAs	Gastric cancer
miR−23a−3p/ACSL4	Epigenetic regulation of ferroptosis via ETS1/miR−23a−3p/ACSL4 axis mediates sorafenib resistance in human hepatocellular carcinoma	Non-coding RNAs	Hepatocellular carcinoma
miR−137/SLC1A5	miR−137 regulates ferroptosis by targeting glutamine transporter SLC1A5 in melanoma	Non-coding RNAs	Melanoma
YTHDF1/PD-L1	N6-methyladenosine regulator YTHDF1 represses the CD8 + T cell-mediated antitumor immunity and ferroptosis in prostate cancer via m6A/PD-L1 manner	m6A modification	miR−3173−5p/ACSL4
FTO/SLC7A11	FTO Prevents Thyroid Cancer Progression by SLC7A11 m6A Methylation in a Ferroptosis-Dependent Manner	m6A modification	Thyroid Cancer
METTL3/SLC7A11	RNA binding protein NKAP protects glioblastoma cells from ferroptosis by promoting SLC7A11 mRNA splicing in an m6A-dependent manner	m6A modification	Glioblastoma
METTL3/SLC7A11	The N6-methyladenosine modification enhances ferroptosis resistance through inhibiting SLC7A11 mRNA deadenylation in hepatoblastoma	m6A modification	Hepatoblastoma
METTL3/SLC7A11	METTL3 promotes lung adenocarcinoma tumor growth and inhibits ferroptosis by stabilizing SLC7A11 m6A modification	m6A modification	Lung adenocarcinoma
YTHDC2/SLC7A11/ SLC3A2	Targeting SLC3A2 subunit of system XC- is essential for m6A reader YTHDC2 to be an endogenous ferroptosis inducer in lung adenocarcinoma	m6A modification	Lung adenocarcinoma
METTL3/FSP1	Exosomal miR−4443 promotes cisplatin resistance in non-small cell lung carcinoma by regulating FSP1 m6A modification-mediated ferroptosis	m6A modification	Non-small cell lung carcinoma
METTL14/SLC7A11/ FPN1	N6-methyladenosine regulated FGFR4 attenuates ferroptotic cell death in recalcitrant HER2-positive breast cancer	m6A modification	Breast cancer
YTHDF2/CBS/ACSL4	Hypoxia inducible lncRNA-CBSLR modulates ferroptosis through m6A-YTHDF2-dependent modulation of CBS in gastric cancer	m6A modification	Gastric cancer
ALKBH5/GPX4/ SLC7A11	ALKBH5 inhibits thyroid cancer progression by promoting ferroptosis through TIAM1-Nrf2/HO−1 axis	m6A modification	Thyroid cancer

TNBC has a heterogeneous phenotype in ferroptosis-related metabolic pathways [53, 54]. The inhibition of GPX4 not only induces tumor ferroptosis, but also enhances anti-tumor immunity. A study integrating multi-omics data from a large TNBC cohort found that TNBC is heterogeneous in ferroptosis-related metabolic pathways. TNBC is characterized by up-regulation of glutathione metabolism (particularly GPX4). Combination therapy of GPX4 inhibitor and anti-PD1 agent has been shown to be more effective than monotherapy [55]. At the same time, cysteine protease inhibitor CST1 can affect the progression of GC by regulating GPX4 and ferroptosis. High expression of CST1 inhibits the ubiquitin modification of GPX4 and ferroptosis, ultimately leading to poor prognosis of GC [56]. Tagitinin C induces endoplasmic reticulum stress and oxidative stress, which activates Nrf2, and then in turn activates the expression of HO-1. Up-regulation of HO-1 leads to an increase of Fe^2+^, which promotes lipid peroxidation and ultimately induces ferroptosis in colorectal cancer cells, and Tagitinin C exerts antitumor effects [57]. In conclusion, ferroptosis may play an anti-tumor role in different tumors, and the epigenetic regulation mechanism of ferroptosis has received more and more attention.

### Cuproptosis

**Fig. 4:**
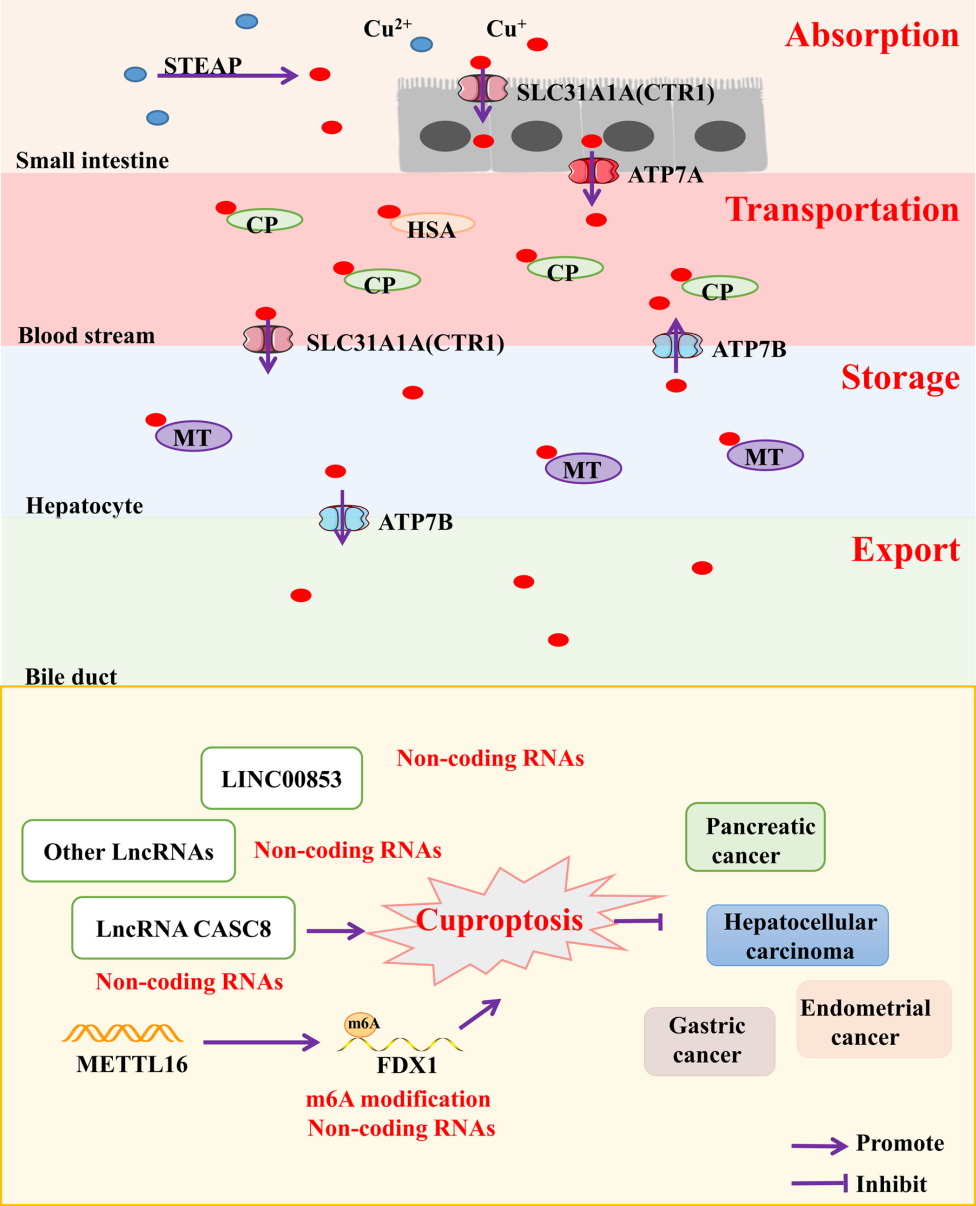
Metabolic processes of Cu + in the human body. Cu^+^ is absorbed in the small intestine, transported to the blood via ATPase copper transporter alpha (ATP7A) and bound to ceruloplasmin (CP), then transferred to the liver via copper transporter 1 (CTR1) and stored in the liver bound to metallothionein (MT), then can be transported to the blood via ATPase copper transport beta (ATP7B) or excreted into the bile

Recently, a novel form of copper-induced cell death has been proposed. Cuproptosis, highly associated with mitochondrial respiration and the lipoic acid pathway, is involved in tumor signaling pathways [44]. Under normal circumstances, copper in the body is in a dynamic, steady balance through the processes of copper absorption, copper storage, copper transportation and copper export [45] (Fig. 4). In the small intestine, Cu^2+^ is converted to Cu^+^ by STEAP, and Cu^+^ is taken up by epithelial cells through copper transporter 1 (CTR1), also known as solute carrier family 31 member 1 (SLC31A1) [46], and then exported to the blood by ATPase copper transporter alpha (ATP7A) [47]. In blood, Cu^+^ which binds to ceruloplasmin (CP) is transported to the liver via SLC31A1 and stored in the liver by binding to metallothionein 1/2 (MT1/2). Cu^+^ is finally transferred to the blood by ATPase copper transportor beta (ATP7B), and excessive copper is excreted into the bile duct by ATP7B and thus leaves the body [45]. The imbalance of copper homeostasis can lead to the increase of intracellular Cu^+^ concentration and change a series of cell signaling pathways.

Cuproptosis is associated with the occurrence and development of different cancers, including HCC, GC and BC (Table 3). Studies have shown that cuproposis can regulate TME, especially change the number of CD8 + T cells, thereby affecting the prognosis of BC patients and their response to immunotherapy [48, 49]. In addition, cuproptosis, together with apoptosis, pyroptosis, and ferroptosis, regulates TME and affects tumor immunity [50]. Lu et al. [51]found that a copper-based nanomedicine has a low reduction of GSH, and it could induce cuproposis in A549/DDP cells and inhibit cisplatin resistance in NSCLC. Xu et al [52] reported a nanomaterial, which can induce cuproptosis of BC. The nanomaterial inhibits tumor growth by reducing intracellular glucose and GSH, and enhancing intracellular H_2_O_2_. Finally, it improves the photodynamic therapy (PDT) efficacy. In addition, a novel prognostic model for hepatocellular carcinoma (LIHC) has been developed based on genes associated with cuproptosis, suggesting that LIPT1 may promote the proliferation, invasion, and migration of LIHC cells. LIPT1 may be a new potential target for the treatment of LIHC [53]. However, there are few literatures on regulatory mechanism of cuproptosis in tumors up to now.

**Table 3:** Epigenetic regulation of tumor cell cuproptosis

Gene	Title	Epigenetic modulation	Cancer
LIPT1/DLAT/ MTF1/GLS…	A novel Cuproptosis-related LncRNA signature to predict prognosis in hepatocellular carcinoma	Non-coding RNAs	Hepatocellular carcinoma
LINC01150	A novel cuproptosis-related lncRNA nomogram to improve the prognosis prediction of gastric cancer	Non-coding RNAs	Gastric cancer
LncRNA AL645608.6/AL591767…	A novel signature to guide osteosarcoma prognosis and immune microenvironment: Cuproptosis-related lncRNA	Non-coding RNAs	Osteosarcoma
RNF139-AS1/LINC00996 NR2F2-AS1…	A novel cuproptosis-related lncRNA signature predicts the prognosis and immune landscape in bladder cancer	Non-coding RNAs	Bladder cancer
CASC8	Analysis of cuproptosis-related lncRNA signature for predicting prognosis and tumor immune microenvironment in pancreatic cancer	Non-coding RNAs	Pancreatic cancer
Cuproptosis-associated lncRNAs	Cuproptosis-related LncRNA signatures as a prognostic model for head and neck squamous cell carcinoma	Non-coding RNAs	Head and neck squamous cell carcinoma
LINC00853	Systemic Analyses of Cuproptosis-Related lncRNAs in Pancreatic Adenocarcinoma, with a Focus on the Molecular Mechanism of LINC00853	Non-coding RNAs	Pancreatic Adenocarcinoma
RAB11B-AS1/AC007552.2/AL035530.2…	Prognostic signature construction and immunotherapy response analysis for Uterine Corpus Endometrial Carcinoma based on cuproptosis-related lncRNAs	Non-coding RNAs	Uterine corpus endometrial carcinoma
AC004112.1/AC007064.2/ AC012186.2…	A novel cuproptosis-related lncRNA signature predicts the prognosis and immunotherapy for hepatocellular carcinoma	Non-coding RNAs	Hepatocellular carcinoma
METTL16/FDX1	Lactylation of METTL16 promotes cuproptosis via m6A-modification on FDX1 mRNA in gastric cancer	m6A modification	Gastric cancer

### Disulfidptosis

**Fig. 5:**
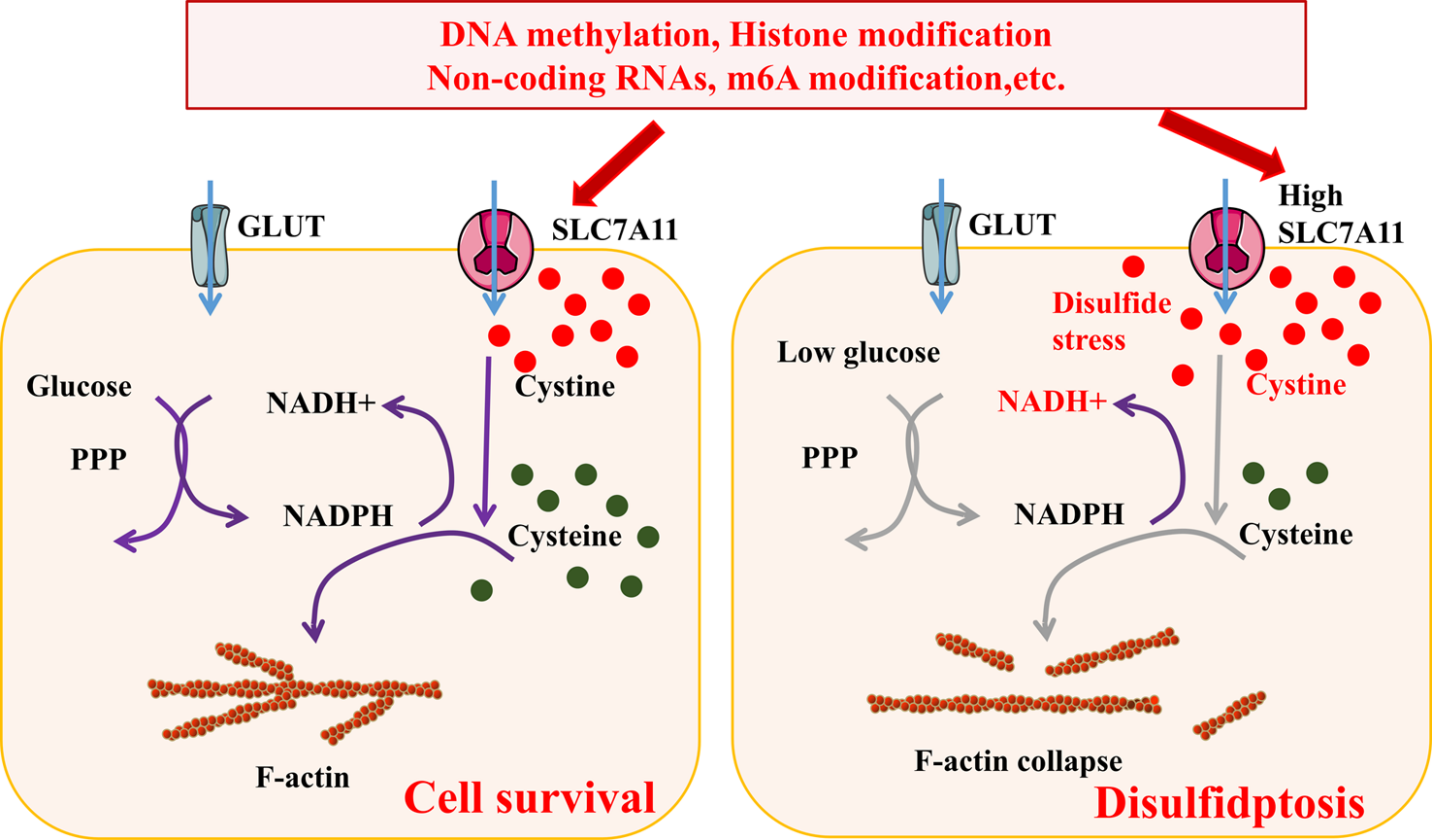
Mechanisms of cellular disulfidptosis. Glucose starvation and high levels of SLC7A11 resulted in cellular NADPH depletion and cystine accumulation, which further led to F-actin collapse and ultimately induced cellular disulfidptosis. *GLUT* Glucose transporter, *PPP* Pentose phosphate pathway, *SLC7A11* Solute carrier family 7 member 11, *NADPH* nicotinamide adenine dinucleotide phosphate. Interconnections between cell death

Interestingly, disulfidptosis, a new form of cell death characterized by the collapse of cytoskeleton proteins, has been reported recently [54, 55]. In disulfidptosis, the depletion of NADPH increases cystine in SLC7A11-overexpressing cancer cells, which eventually leads to the accumulation of intracellular disulfide molecules and cell death [54, 56]. NADPH depletion and accumulation of disulfides causes disulfide stress, and then this stress activates some signaling pathways, leading to aberrant disulphide bonds and disulfidptosis in actin cytoskeleton [6]. In conclusion, NADPH synthesis is reduced when the cell is in glucose-starvation. High expression of SLC7A11 results in NADPH depletion and increases NADH^+^. Cystine cannot be decomposed into cysteine due to the decrease of NADPH. Excessive cystine content eventually induces disulfide stress (Fig. 5).

One study which analyzed the genetic profile of HCC patients found that mutations in disulfidptosis related genes occurred in 7.14% of patients, and the patients with more disulfidptosis had a higher degree of immune infiltration and immunosuppression. New biomarkers associated with disulfidptosis can be used to predict prognosis and therapeutic target in clinical diagnosis and treatment of HCC [57]. In addition, BC patients with higher scores of disulfidptosis-related genes have a lower survival, higher inflammation rate in the TME, and increased tumor mutation burden. The expression of disulfidptosis related gene CTSE is increased in BC tissues, which promotes the proliferation and metastasis of BC cells [58]. In addition, the disulfidptosis score can predict the prognosis of cervical cancer [59], breast cancer [60], lung adenocarcinoma [61], thyroid cancer [62] and renal cell carcinoma [63].

### Interconnections between cell death

**Fig. 6:**
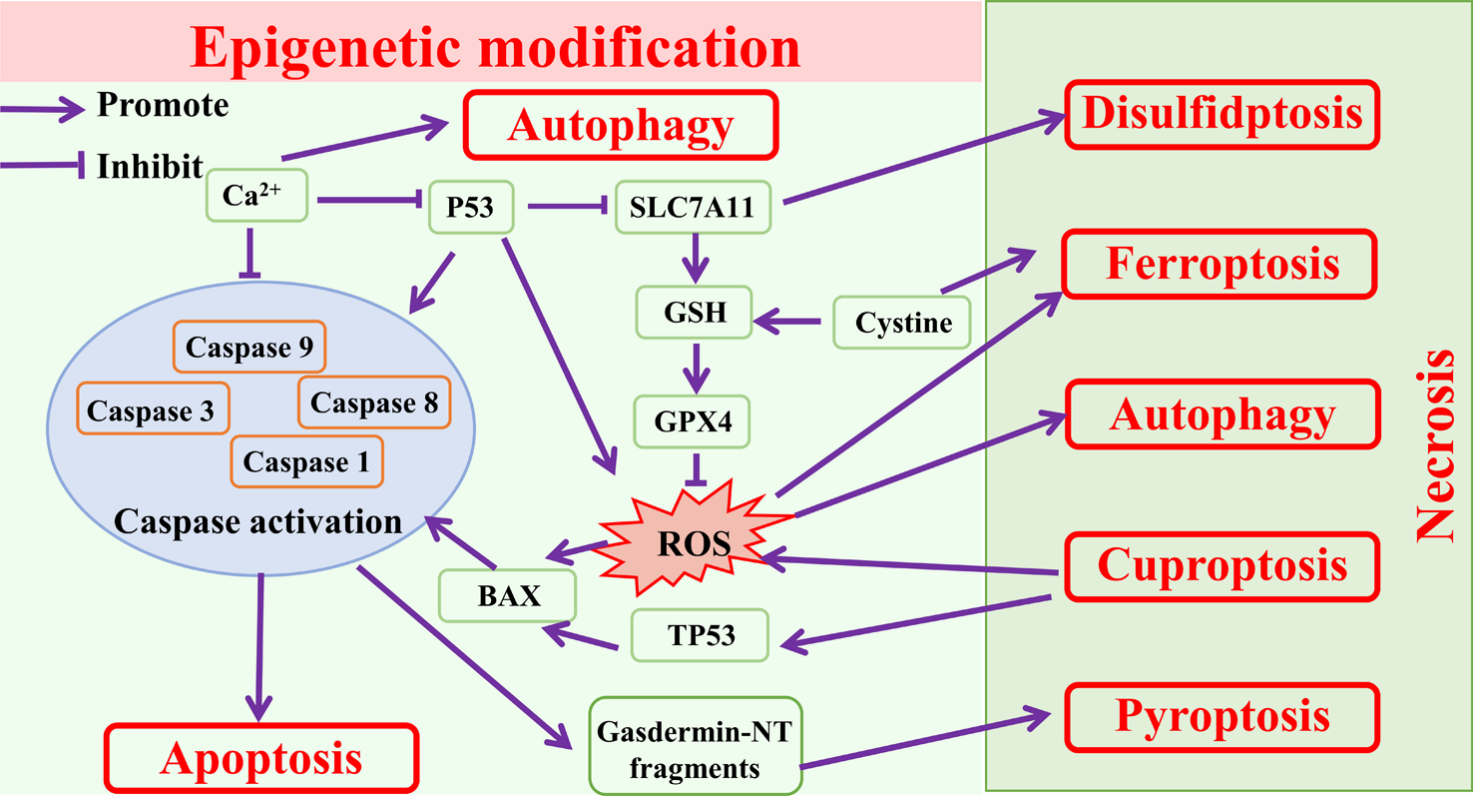
Interconnections between cell death. *SLC7A11* Solute carrier family 7 member 11, *GPX4* glutathione peroxidase 4, *GSH* Glutathione, *BAX* BCL2 associated X, apoptosis regulator, *TP53* Tumor protein p53

The interconnections between various modes of cell death are illustrated in Fig. 6. Firstly, key molecules are shared between different modes of cell death. SLC7A11 not only inhibits ferroptosis by up-regulating GPX4 expression and reducing lipid peroxidation [64], but also promotes disulfidptosis by enhancing NADPH depletion [54]. Reactive oxygen species (ROS) is involved in the regulation of various cell deaths, and excessive ROS promotes cellular ferroptosis [65], autophagy [66], pyroptosis [67] and apoptosis [68]. Ca^2+^ up-regulation promotes autophagy and inhibits apoptosis [69, 70]. Secondly, one mode of cell death can regulate the onset of another mode of cell death. Cellular cuproptosis promotes ROS production, which in turn regulates other cell deaths, and excessive Cu^+^ induces TP53-dependent apoptosis through activation of TP53 target genes such as BAX [71].Human umbilical cord mesenchymal stem cells-derived exosomes inhibit cellular pyroptosis by increasing autophagy levels through miR-146a-5p/TRAF6 [72]. Thirdly, the same stimulus can induce different forms of cell death, depending on the stimulus intensity alongside other factors. P53 promotes apoptosis [73] and ferroptosis [74, 75]. SLC7A11 inhibits ferroptosis [76] and promotes disulfidptosis [54]. Ca^2+^ up-regulation promotes autophagy and inhibits apoptosis [70]. Finally, multiple modes of cell death can act together to regulate the progression of the same disease, i.e., ferroptosis, autophagy, pyroptosis and apoptosis are all involved in regulating renal disease [7]. Since different modes of cell death share a number of key molecules, epigenetic modifications on those molecules can affect various cell death.

DNA methylation, histone modification and non-coding RNAs modification affect various patterns of cell death [77]. DNA methylation can modulate autophagy and tumour growth by regulating the expression of autophagy genes such as Beclin-1, ATG4A and ELFN2. In addition, histone acetylation and dysregulation of non-coding RNAs mediated autophagy have been associated with different kinds of cancers [77, 78]. DNA methylation and histone phosphorylation regulate apoptosis by modulating the expression of apoptosis-related genes including P53, BAX and Caspase-8 [79]. DNA methylation regulates ferroptosis-related genes such as SCD1 and FADS2 to suppress ferroptosis and histone acetyltransferases inhibit ferroptosis by regulating GSH production [77]. The lymphocyte-specific deconjugation enzymes, which modify P53 via chromatin modification, histones and non-coding RNAs all affect expression of SLC7A11, thus induce ferroptosis [80], even the possibility of regulating disulfidptosis is further suspected.

## Epigenetic modifications in different cell death modes

### Epigenetic modifications in pyroptosis

Common epigenetic modifications include DNA methylation, m6A modification, and histone modification. Epigenetic modifications can affect the development and prognosis of cancer by regulating pyroptosis (Table 1). There are five major inflammasomes in the pyroptosis pathway: NLRP3, AIM2, NLRP1, PYRIN, and NLRC4. The inflammasomes activate caspase family (caspase 1, caspase 3, etc.) and Gasdermin family (such as GSDMD, usually acting on the N-terminal domain of GSDMD), thus induce pyroptosis (Fig. 2).

DNA methylation, one of the epigenetic modifications, can regulate pyroptosis of tumor cells. Studies have shown that DNA methylation mediates the down-regulation of ZDHHC1 in cancer. Up-regulation of ZDHHC1 can alter cellular metabolism and induce oxidative stress-mediated pyroptosis to inhibit tumor growth [81]. Decitabine up-regulates the expression of GSDME by inhibiting DNA methylation, and then causes pyroptosis of cancer cells and induces anti-tumor immunity of melanoma and TNBC [82, 83]. miR-495 promoter methylation can down-regulate miR-495, and the overexpressed miR-495 can attenuate NLRP3 activation to alleviate macrophage inflammation and pyroptosis [84].

m6A modification can also regulate pyroptosis. Interestingly, up-regulation of H3K4me1 and H3K27Ac promotes lncRNA LINC00969 expression in NSCLC cells, then LINC00969 interacts with METTL3 and inhibits NLRP3 in an m6A-YTHDF2-dependent manner to inhibit pyroptosis in NSCLC, thereby promoting drug resistance of NSCLC [85]. Tanshinone IIA enhances pyroptosis and inhibits HK1 cell proliferation by regulating miR-125b/foxp3/caspase-1/GSDMD signaling, providing strong evidence for the treatment of nasopharyngeal carcinoma [86]. YTHDC1 down-regulates lncRNA FENDRR stability and regulates the caspase-1/NLRP3 pathway through DRP1 DNA methylation to modulate the pyroptosis of hypoxia-induced pulmonary artery endothelial cells [87]. Simvastatin in combination with DNA methyltransferase inhibitors inhibits GC through caspase-3/GSDME-mediated pyroptosis. It is suggested that inducing cancer cell-specific pyroptosis by activating GSDME expression may be a potential therapeutic strategy against GC [88]. In addition, METTL14 up-regulates the m6A methylation level of TINCR gene and YTHDF2 promotes the degradation of TINCR gene, leading to the decrease of the expression of TINCR. As a result, the positive regulation of NLRP3 by TINCR is reduced, which ultimately promotes pyroptosis and regulates diabetic cardiomyopathy [89]. Furthermore, down-regulation of METTL14 reduces its binding to the m6A site of NEAT1 and subsequently promotes NEAT1 expression through YTHDC1 recognition. In addition, NEAT1 promotes transcriptional activation of NLRP3 by binding to KLF4 (Kruppel-like factor 4), which ultimately promotes the pyroptosis of endothelial cells [90]. However, in the tissues of renal cell carcinoma (RCC), METTL14 regulates the expression of NEAT1 through another recognition protein YTHDF2. Down-regulation of METTL14 is associated with malignant features of RCC, indicating a poor prognosis [91].

### Epigenetic modifications in ferroptosis

Studies have revealed that DNA methylation, microRNA, m6A modification and other epigenetic ways can regulate the differential expression of most ferroptosis-related genes in tumors [92, 93](Table 2). Ferroptosis is a mode of cell death caused by increased levels of cellular Fe^2+^ leading to significantly increased levels of cellular lipid peroxidation. Ferroptosis-related proteins include GPX4, ACSL4, SCL7A11, FTH1, and FSP1 (Fig. 3).

Recent data from The Cancer Genome Atlas (TCGA) show that multiple ferroptosis-related genes are dysregulated in cancer, which may be related to aberrant DNA methylation [94]. Overexpression of SLC7A11, a transporter protein that inputs cysteine for the biosynthesis of glutathione and causes antioxidant defense, inhibits ferroptosis and promotes tumor growth [64, 95]. H3K9 demethylase KDM3B reduces the methylation of H3K9 in the promoter of SLC7A11 and promotes the transcription of SLC7A11. On the contrary, BAP1 and PRC1 deubiquitinate H2A in the promoter of SLC7A11 and inhibit the transcription of SLC7A11 [95]. Lymph specific helicase (LSH) is a chromatin remodeling enzyme. DNA methylation modification and lncRNA modification can up-regulate the expression of LSH and reduce the expression of ferroptosis-related genes to regulate ferroptosis in lung cancer and leukemia [96, 97].

Another common epigenetic modification, m6A modification, is also conducive to ferroptosis in tumor. CBSLR, a hypoxia-induced lncRNA, reduces CBS mRNA stability by YTHDF2, leads to the reduction of ACSL4 protein methylation and ACSL4 degradation, and ultimately inhibits the ferroptosis of GC cells, enhances chemoresistance and affects prognosis [98]. In addition, YTHDF1, which is upregulated in prostate cancer samples, promotes PD-L1 expression in an m6A-PD-L1 manner, reduces effector T-cell toxicity and CD8 + T-cell-mediated immunosuppression and ferroptosis, and finally leads to poor clinical outcomes [99]. In hepatoblastoma (HB), highly up-regulated SLC7A11 promotes the proliferation of HB cells in vitro and in vivo, and inhibits the ferroptosis in HB cells. METTL3 promotes SLC7A11 expression by IGF2BP1-m6A modification. IGF2BP1 inhibits SLC7A11 degradation by blocking BTG4/CCR1-NOT complex recruitment through competitive binding to PABPC2 [100]. Interestingly, hypoxia inhibits METTL14 expression in a HIF-1α-dependent manner, which in turn reduces m6A methylation of the SLC7A11 mRNA 5’UTR in HCC and up-regulates SLC7A11 expression via the YTHDF2 pathway to inhibit ferroptosis in HCC [101]. In conclusion, multiple epigenetic modalities regulate ferroptosis of cancer cells.

### Epigenetic modifications in cuproptosis

The cuproptosis is a new concept in the past two years and there are few studies on the epigenetic mechanism of cuproptosis in tumors. Fortunately, the lncRNA modifications in epigenetic regulatory modifications have been reported more frequently (Fig. 4; Table 3).

The cuproptosis-related lncRNA LINC00853 significantly enhances cellular glycolysis and cell proliferation in pancreatic cancer (PC) cells by PFKFB3 and increases the level of cellular mitochondrial respiration and tumor growth rate [102]. Meanwhile, another cuproptosis-related lncRNA, CASC8, is highly expressed in PC, making the prognosis of PC worse. Knockdown of CASC8 gene inhibits the proliferation and migration of PC cells and reduces the expression of CD274, CXCL10, CXCL11 and CXCL9, which affects the TME [103]. In addition, cuproptosis-related lncRNA markers are also differentially expressed in HCC [104], BC [105], and endometrial carcinoma [106], which can predict prognosis. Interestingly, copper stress promotes lactate modification at the K229 site of METTL16, which in turn increases the level of m6A modification of FDX1 to promote FDX1 expression, ultimately inducing cuproptosis in GC [107]. Since there have been few studies on the regulation of tumor cuproptosis by m6A modification in recent years, the regulation of tumor cuproptosis by m6A modification may become a new research hotspot in the future.

### Epigenetic modifications in disulfidptosis

Although some studies have reported that disulfidptosis affects the prognosis of various tumors and tumor immune infiltration, the epigenetic modifications mechanism of disulfidptosis is not completely clear. Interestingly, studies have shown that disulfidptosis-related lncRNAs in breast cancer can accurately predict breast cancer subtypes. In the basal subtype, LINC02188 has the highest expression while LINC01488 and GATA3-AS1 have the lowest. In the Her2-positive subtype, LINC00511 has the highest expression. In the Luminal A and Luminal B subtype, GATA3-AS1 has the highest expression, while LINC00511 has the lowest expression. In addition, key lncRNAs are closely related to RNA methylation modification and angiogenesis [108]. Furthermore, disulfidptosis-related lncRNAs ZSCAN16-AS1, AC083799.1, AL021707.6, and LINC02356 could be used as protective factors, while AC023043.1 could be used as a risk factor in cervical cancer patients. The patients with high disulfidptosis-related lncRNA index have showed increased sensitivity to immunotherapy [59]. Unfortunately, there are few studies examining the mechanisms of other epigenetic modifications on the regulation of disulfidptosis.

### Epigenetic regulation of cell death proteins

Epigenetic regulation of different cell death modes is mainly involved in the regulation of key cell death proteins and affects cell death by up-regulating or down-regulating their expressions. In conclusion, epigenetic modifications promotes or inhibits pyroptosis of tumor cells by regulating DNA methylation, RNA methylation and histone modification of key pyroptosis-related proteins such as caspase-1, caspase-3, GSDMA/B/D/E, NLRP1 and NLRP3, which in turn affects the regulation of tumor progression and drug resistance. In addition, epigenetic modifications regulate ferroptosis in tumor cells by modulating key ferroptosis-related proteins, such as GPX4, ACSL4, SCL7A11, FTH1, FSP1. However, epigenetic modifications regulate tumor cuproptosis and disulfidptosis by modulating the expression of cuproptosis and disulfidptosis-associated lncRNAs. Currently, epigenetic modifications such as DNA methylation, RNA methylation and histone modification on tumor cuproptosis and disulfidptosis have not been adequately explored, requiring further research.

Besides m6A modification, another two of RNA modifications, m5C and m7G modification are less studied at present. The study found that m5C methylation modification-related genes were significantly correlated with the incidence of osteoarthritis and rheumatoid arthritis, regarded as a new potential diagnostic biomarker [109]. NSUN2 activates the m5C modification of SLC7A11 mRNA and YBX1 directly binds to SLC7A11 mRNA to promote SLC7A11 expression. The NSUN2/SLC7A11 axis inhibits endometrial cancer (EC) growth by increasing ferroptosis, suggesting that NSUN2 may serve as a prognostic biomarker and therapeutic target for EC patients [110]. METTL1 decreases FTH 1 expression by enhancing m7G modification of FTH 1 and pri-miR-26a, and thus increase ferroptosis, thereby reducing chemoresistance of osteosarcoma, so targeted therapy on METTL1 may be a potential and promising treatment strategy for osteosarcoma [111]. However, currently it is difficult to study m5C and m7G modifications on cuproptosis and disulfidptosis, which may become a hotspot in the future.

## Targeting cell death pathways for cancer therapy

### DNMT inhibitors

Different cells in tumor tissues exhibit different epigenetic modification patterns throughout the genome or at individual genes, and the reversibility of aberrant DNA methylation and acetylation is a common target for cancer therapy. Therefore, many small molecule inhibitors targeting chromatin and histone modifying enzymes have emerged clinically as anticancer agents, such as DNA methyltransferase (DNMT) inhibitors and histone deacetylase (HDAC) inhibitors. DNMT inhibitors includes 5-azacytidine and decitabine (5-aza-2’-deoxycytidine, Aza); HDAC inhibitors include vorinostat (SAHA), romidepsin (depsipeptide), tricostatin A (TSA) [112].

DNMT inhibitors, such as 5-azacytidine and decitabine, exert their demethylation activity [113]. Decitabine inhibits the expression of human hyaluronidase family PH20 by activating the lncRNA PAS1, leading to the regression on breast cancer growth and metastasis [114]. Furthermore, decitabine promotes sensitivity to platinum-based therapy in patients with ovarian cancer, indicating a favorable prognosis [115]. Besides, decitabine promotes increased neoantigen presentation of MHC class I in glioblastoma cells, leading to increased activation of specific T-cells to promote their killing of cancer cells [116]. The DNA methylation inhibitor 5-azacytidine effectively reverses the expression of prostate cancer suppressor genes and cell growth in vitro and in vivo, and enhances the anticancer efficacy of docetaxel [117]. In addition, the combination of two DNMT inhibitors is more effective in suppressing tumors.

### HDAC inhibitor

One potential target for cancer treatment is a group of epigenetic regulators known as HDAC inhibitors. These inhibitors remove acetyl groups from both histones and non-histone proteins, ultimately down-regulating the transcription of genes [115]. The FDA has approved the use of HDAC inhibitors including SAHA, romidepsin (depsipeptide), and belinostat for certain cancer such as T-cell lymphoma. Panobinostat (LBH 589) is approved for the treatment of multiple myeloma [118]. Besides, sulforaphane is a class of HDAC inhibitors used in the treatment of colorectal cancer, which can inhibit tumor progression by targeting HDAC1 and HDAC2 [119]. Vorinostat is another orally available HDAC inhibitors that inhibits HDAC I and II; it also acts indirectly under hypoxic conditions by inhibiting hypoxia-inducible factor (HIF)-1α and vascular endothelial growth factor (VEGF), ultimately blocking angiogenesis [113]. The combination of DNMT inhibitors and HDAC inhibitors is frequently used in the clinic for the treatment of cancers such as gastrointestinal tumors and melanoma [113, 120].

The combination of Aza and TSA can attenuate inflammation-induced pyroptosis during endotoxaemia-induced acute lung injury (ALI). The combination of Aza and TSA inhibits LPS-induced cellular pyroptosis in BMDMs by inhibiting the JNK-ERK and STAT3-JMJD3 pathways, leading to lower levels of DNA fragmentation and reduced transcriptional and translational expression of pyroptosis-related genes [121]. Therefore, combined use of Aza and TSA targeting these important signaling pathways would be a good therapeutic approach for ALI. In addition, Aza reduces DNMT1 methylation of lncRNA GAS5 to inhibit the NLRP3 axis and cardiac fibroblast pyroptosis, considered as a potential therapeutic target for cardiac fibrosis [122]. Furthermore, Aza inhibits DNMT-1 and then alleviates ferroptosis through NCOA4 mediated ferritinophagy during diabetic myocardial ischemia/reperfusion injury [123]. Therefore, it is important to investigate the mechanism of diabetes-induced myocardial injury for the prevention and treatment of myocardial injury.

### Others

In recent years, a number of drugs targeting cancer cell death have emerged (Table 4). FL118, a novel camptothecin anticancer drug, is found to inhibit the growth and metastasis of colorectal cancer through the NLRP3-ASC-Caspase-1 pathway by mediating the pyroptosis of SW480 and HT29 cells. FL118 induces pyroptosis mediated by the NLRP3 inhibitor MCC950 and the caspase-1 inhibitor VX-765 attenuate the expression of NLRP3 and caspase-1 in colorectal cancer cells [124]. FL118 shows the antitumor activity and resistance superior to its clinical analogues irinotecan and topotecan in the colorectal and lung tumor transplantation model of thymus-free mice [124], revealing FL118 as a potential anticancer agent worthy of further development. Caspase-1, IL-1β, and IL-18 are down-regulated in NSCLC tumor tissues, and simvastatin activates NLRP3-caspase-1-IL-1β and IL-18 pathways to induce pyroptosis and inhibit NSCLC cell migration. The addition of Ac-YVAD-CMK, a specific caspase-1 inhibitor, reduces the growth inhibition of H1229 and A549 cells mediated by simvastatin [125]. Trichosanthin (TCS) promotes apoptosis by up-regulating NLRP3, ASC, caspase-1 and GSDMD to inhibit the proliferation, migration and invasion of NSCLC cells [126]. TCS has been shown to have inhibitory effects on different types of tumors, including NSCLC, and TCS can inhibit NSCLC progression by promoting focal death. These findings provide further information on the possible underlying mechanism of TCS in the treatment of NSCLC. In addition, a self-assembling nanotoxin that can be used to target CXCR4 expression activates melanoma pyroptosis through the caspase-3-GSDME pathway in melanoma [127].

**Table 4:** Targeting cell death pathways for cancer therapy

Drugs	Title	Pathways	Cancer	Death
FL118	Pyroptosis is involved in the inhibitory effect of FL118 on growth and metastasis in colorectal cancer	NLRP3-ASC-Caspase-1	Colorectal cancer	Pyroptosis
Simvastatin	Simvastatin Suppresses Proliferation and Migration in Non-small Cell Lung Cancer via Pyroptosis	NLRP3-caspase-1-IL-1β and IL-18	Non-small cell lung cancer	Pyroptosis
Nanotoxin	A self-assembling CXCR4-targeted pyroptosis nanotoxin for melanoma therapy	CXCR4-caspase-3-GSDME	Melanoma	Pyroptosis
Erastin	Nedd4 ubiquitylates VDAC2/3 to suppress erastin-induced ferroptosis in melanoma	FOXM1-Nedd4-VDAC2/3	Melanoma	Ferroptosis
Erastin	CAF secreted miR−522 suppresses ferroptosis and promotes acquired chemo-resistance in gastric cancer	Exo-miR−522 and ALOX15	Gastric cancer	Ferroptosis
Erastin	Deubiquitinase USP35 modulates ferroptosis in lung cancer via targeting ferroportin	USP35-FPN	Lung cancer	Ferroptosis
Sorafenib and erastin	Ferroptosis inducers enhanced cuproptosis induced by copper ionophores in primary liver cancer	FDX1-GSH	Liver cancer	Ferroptosis
Sulfasalazine	CISD2 inhibition overcomes resistance to sulfasalazine-induced ferroptotic cell death in head and neck cancer	CISD2 -lipid ROS	Neck cancer	Ferroptosis
Sorafenib	Increased ATF2 expression predicts poor prognosis and inhibits sorafenib-induced ferroptosis in gastric cancer	ATF2-HSPH1-SL7A11	Gastric cancer	Ferroptosis
Bortezomib	The cuproptosis-related signature associated with the tumor environment and prognosis of patients with glioma	IGFBP2	Glioma	Cuproptosis
Trientine tetrahydrochloride, penicillamine	Trientine tetrahydrochloride versus penicillamine for maintenance therapy in Wilson disease (CHELATE): a randomised, open-label, non-inferiority, phase 3 trial	Inhibitors of acetyltransferases, copper ion concentration	Wilson disease	Cuproptosis
Triethylenetetramine (trientine)	Triethylenetetramine (trientine): a caloric restriction mimetic with a new mode of action	Inhibitors of acetyltransferases, copper ion concentration	Wilson disease	Cuproptosis
GOx@[Cu(tz)]	An Enzyme-Engineered Nonporous Copper(I) Coordination Polymer Nanoplatform for Cuproptosis-Based Synergistic Cancer Therapy	SAT1-AcCoA	Bladder cancer	Cuproptosis

Ferroptosis inducers (FIN) have shown great potential in cancer therapy. Erastin is the most common inducer of ferroptosis in tumors, including melanoma [128], GC [129], lung cancer [130], and liver cancer [131], by inhibiting the expression of SLC7A11 to limit intracellular cysteine and glutathione levels. Sulfasalazine, an FDA-approved anti-inflammatory agent with FIN activity, can be used to treat head and neck cancer by inducing ferroptosis [132]. Sorafenib, another clinically approved drug inhibiting SLC7A11, can be used for GC [133], liver cancer [134–136], etc. Moreover, another type of FIN can induce ferroptosis by blocking the enzymatic activity of GPX4, such as RSL3, ML162, and ML210, thus achieving the inhibition of cancer [137, 138].

In recent years, researchers have begun to pay attention to the role of cuproposis-related genes in predicting the efficacy of cancer chemotherapy. Studies have found that bortezomib induces cuproposis and enhances efficacy in different cancer risk groups [139]. Various cancer therapeutic drugs can induce or inhibit cuproposis by increasing or decreasing intracellular copper ion concentration, such as penicillamine [140] and trientine [141]. Interestingly, a novel Cu-based nanomaterial, GOx @ [Cu (tz)], significantly induces cuproposis to inhibit BC in mice [142]. There is a significant correlation between cuproposis and the level of mitochondrial metabolism. Copper ionophore combined with anti-tumor drugs can be used to treat high mitochondrial metabolism tumors [45]. In addition to copper ionophores, copper complexes can also increase the concentration of copper ions in cells, leading to cancer cell death [143].

Studies on disulfidptosis are still relatively scarce, and further research is needed to understand its pathogenesis and potential pathophysiological functions. The techniques currently available for inducing disulfidptosis are limited. Future anti-tumor therapies could target depletion of intracellular NADPH and direct induction of disulfide bond stress to effectively induce disulfidptosis.

## Conclusion and perspective

In summary, DNA methylation, histone modification, m6A modification and other epigenetic modifications play a role in regulating different death modes of tumor cells, such as pyroptosis, ferroptosis and cuproptosis. Tumorigenesis is a complex system involving the activation of multiple signaling pathways, changes in TME, and the regulation of various cell death modes. Cancer treatment is more complicated. Besides traditional treatment such as surgery, radiotherapy and chemotherapy, more options such as immunotherapy, targeted therapy and gene therapy are emerging. Therefore, up-regulation or down-regulation of some epigenetic modification-related genes can enhance the sensitivity of tumors to treatment. Understanding the key role of epigenetic modifications in drug resistance of cancer provides new perspectives for the development of drugs or combination strategy.

Clinically histone-modifying drugs are widely used in cancer therapy, including HDAC inhibitors and KAT inhibitors [144]. Histone modifications are classified as writers, readers and erasers. Histone modification writers include lysine methyltransferases (KMTs), protein arginine methyltransferases (PRMTs), lysine acetyltransferases (KATs or HATs), histone ubiquitin ligases, histone kinases [145]. Histone modification readers include chromodomain, Tudor domain, MBT domain, PhD finger, bromodomain-containing proteins [146]. Histone modification erasers are lysine demethylases (KDMs), histone deacetylases (HDACs and SIRTs), histone deubiquitinating enzymes, and histone phosphatases [144, 147]. Therefore, the in-depth understanding of histone modification mechanism in different cell death modalities could provide references for the development of more targeted drugs.

Epigenetic modifications play a role in regulating pyroptosis, ferroptosis and cuproptosis of tumor cells. However, there are few studies on epigenetic modifications of disulfidptosis, and only some studies imply that disulfidptosis-related lncRNAs can predict the prognosis of tumors, but the mechanism is not clear yet. Meanwhile, the interaction between different cell death modalities and the crosstalk between tumor cells and non-tumor cells in TME need to be further studied.

Interestingly, SLC7A11 is involved in regulating both ferroptosis and disulfidptosis of tumor cells, with a double-edged sword effect, i.e., anti-tumor or tumor-promoting effect. The low expression of SLC7A11 inhibits the conversion of cystine to cysteine, and then inhibits the synthesis of GSH. The decrease of GSH synthesis leads to the decrease of antioxidant capacity, which promotes lipid peroxidation and eventually induces ferroptosis, thus inhibiting tumor growth. However, when glucose starvation leads to NADPH depletion, cystine cannot be converted to cysteine if SLC7A11 is highly expressed. Cystine accumulation results in disulfide stress, inducing disulfidptosis, and then increased disulfidptosis further alters the prognosis of the tumor. The above-mentioned evidences suggest that there must be some connection between different cell death modes, and different expression levels of the same gene can induce different cell death modes, so whether it plays an anti-tumor or a tumor-promoting role should be considered.

### Electronic supplementary material

Below is the link to the electronic supplementary material.

Supplementary Material 1

## Data Availability

No datasets were generated or analysed during the current study.
