# Supplementary material for: Epigenetic regulation of diverse cell death modalities in cancer: a focus on pyroptosis, ferroptosis, cuproptosis, and disulfidptosis
Source: J Hematol Oncol. 2024 Apr 23;17:22. doi: 10.1186/s13045-024-01545-6 (PMC11040947; doi:10.1186/s13045-024-01545-6)
Supplement: Supplementary file 1 — Supplementary Material 1 [file 13045_2024_1545_MOESM1_ESM.docx]

English Abbreviations

| Abbreviations | Full name |
| --- | --- |
| 5'UTR | 5' untranslated regions |
| ACSL4 | Acyl-coenzyme A synthetase long chain family member 4 |
| AIM2 | Absent in melanoma 2 |
| ALKBH5 | AlkB homolog 5, RNA demethylase |
| ATP7A | ATPase copper transporter alpha |
| ATP7B | ATPase copper transport beta |
| BAX | BCL2-associated X protein |
| BC | Bladder cancer |
| BH4 | Tetrahydrobiopterin |
| CASC8 | Cancer susceptibility 8 |
| CCR5 | C-C motif chemokine receptor 5 |
| CoQH2 | Ubiquinol |
| CP | Ceruloplasmin |
| CST1 | Cystatin SN |
| CTR1 | Copper transporter 1 |
| CXCL9/10 /11 | C-X-C motif chemokine ligand 9/10/11 |
| DHODH | Dihydroorotate dehydrogenase |
| DNMTs | DNA methyltransferases |
| DRD2 | Dopamine receptor D2 |
| ECM | Extracellular matrix |
| EMT | Epithelial mesenchymal transformation |
| FSP1 | Ferroptosis suppressor protein-1 |
| FTH1 | Ferritin heavy chain 1 |
| FTL1 | Ferritin light polypeptide 1 |
| FTO | Fat mass and obesity associated |
| GC | Gastric cancer |
| GCH1 | GTP cyclohydroxylase-1 |
| GPX4 | Glutathione peroxidase 4 |
| GSDMD | Gasdermin D |
| GSDME | Gasdermin E |
| GSH | Glutathione |
| HB | Hepatoblastoma |
| HCC | Hepatocellular carcinoma |
| IGF2BP1/2/3 | Insulin like growth factor 2 mRNA binding protein 1/2/3 |
| IL-10 | Interleukin 10 |
| IL-6 | Interleukin 6 |
| KDM3B | Lysine demethylase 3B |
| LIHC | Hepatocellular carcinoma |
| LIPT1 | Lipoyltransferase 1 |
| LPS | Lipopolysaccharide |
| LSH | Lymph specific helicase |
| m5C | N5-methylcytidine |
| m6A | N6-methyladenosine |
| m7G | N7-methylguanosine |
| MDSC | Myeloid-derived suppressor cells |
| MEG3 | Maternally expressed 3 |
| METTL3/14 | Methyltransferase 3/14 |
| MMP | Matrix metalloproteinase |
| MT1/2 | Metallothionein 1/2 |
| NADPH | Nicotinamide adenine dinucleotide phosphate |
| NEAT1 | Nuclear paraspeckle assembly transcript 1 |
| NLRC4 | NLR family CARD domain containing 4 |
| NLRP1 | NLR family pyrin domain containing 1 |
| NLRP3 | NLR family pyrin domain containing 3 |
| NSCLC | Non-small cell lung cancer |
| PCa | Prostate cancer |
| PD-1 | Programmed cell death 1 |
| PGE2 | Prostaglandin E2 receptor EP3 subtype-like |
| PUFA-PL | Polyunsaturated fatty acid-containing phospholipid |
| PYRIN | MEFV innate immunity regulator, pyrin |
| RCC | Renal cell carcinoma |
| RNS | Reactive nitrogen species |
| ROS | Reactive oxygen species |
| SLC31A1 | Solute carrier family 31 member 1 |
| SLC7A11 | Solute carrier family 7 member 11 |
| STAT3 | Signal transducer and activator of transcription 3 |
| TAMs | Tumor-associated macrophages |
| TCGA | The cancer genome atlas |
| TETs | Tet methylcytosine dioxygenases |
| TGFβ | Transforming growth factor beta |
| TLR4 | Toll like receptor 4 |
| TME | The tumor microenvironment |
| TNBC | Triple-negative breast cancer |
| TP53 | Tumor protein p53 |
| VEGF | Vascular endothelial growth factor |
| YTHDC1/2/3 | YTH N6-methyladenosine RNA binding protein C1/2/3 |
| YTHDF1/2/3 | YTH N6-methyladenosine RNA binding protein F1/2/3 |
| ZDHHC1 | Zinc finger DHHC-type containing 1 |
